# Direct observation of surface charge and stiffness of human metaphase chromosomes†

**DOI:** 10.1039/d2na00620k

**Published:** 2022-12-20

**Authors:** Seokbeom Roh, Taeha Lee, Da Yeon Cheong, Yeonjin Kim, Soohwan Oh, Gyudo Lee

**Affiliations:** a Department of Biotechnology and Bioinformatics, Korea University Sejong 30019 Korea lkd0807@korea.ac.kr; b Interdisciplinary Graduate Program for Artificial Intelligence Smart Convergence Technology, Korea University Sejong 30019 Korea; c College of Pharmacy, Korea University Sejong 30019 Korea soohwanoh@korea.ac.kr

## Abstract

Metaphase chromosomes in which both polynucleotides and proteins are condensed with hierarchies are closely related to life phenomena such as cell division, cancer development, and cellular senescence. Nevertheless, their nature is rarely revealed, owing to their structural complexity and technical limitations in analytical methods. In this study, we used surface potential and nanomechanics mapping technology based on atomic force microscopy to measure the surface charge and intrinsic stiffness of metaphase chromosomes. We found that extra materials covering the chromosomes after the extraction process were positively charged. With the covering materials, the chromosomes were positively charged (*ca.* 44.9 ± 16.48 mV) and showed uniform stiffness (*ca.* 6.23 ± 1.98 MPa). In contrast, after getting rid of the extra materials through treatment with RNase and protease, the chromosomes were strongly negatively charged (*ca.* −197.4 ± 77.87 mV) and showed relatively non-uniform and augmented stiffness (*ca.* 36.87 ± 17.56 MPa). The results suggested undulating but compact coordination of condensed chromosomes. Additionally, excessive treatment with RNase and protease could destroy the chromosomal structure, providing an exceptional opportunity for multiscale stiffness mapping of polynucleotides, nucleosomes, chromatin fibers, and chromosomes in a single image. Our approach offers a new horizon in terms of an analytical technique for studying chromosome-related diseases.

## Abstract 2

The biophysical properties of human metaphase chromosomes were directly measured by Kelvin probe force microscopy (KPFM) and PeakForce-quantitative nanomechanics (PF-QNM) with high resolution.

## Graphical abstract

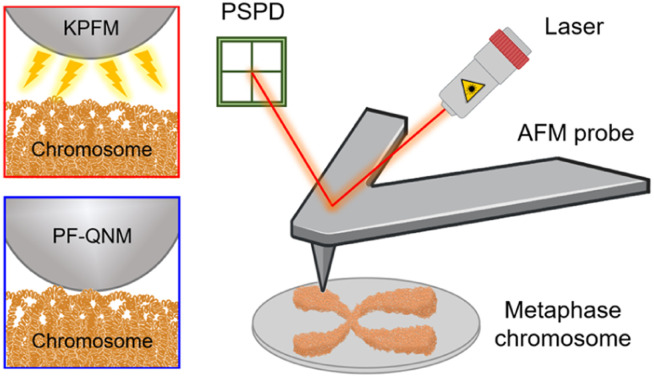

## Introduction

The architecture of mitotic chromosomes in a variety of models from bacteria to eukaryotes has been actively studied.^1–6^ During every single cell cycle in eukaryotic cells, dispersed interphase chromosomes are elaborately condensed and form pairs of sister chromatids with the help of structural maintenance of chromosome (SMC) proteins such as condensin and cohesin.^7–9^ During cell division, the structure and composition of chromosomes rapidly change according to specific stages,^10^ and these changes are closely related to life phenomena such as cell division, cancer development, and cellular senescence.^2,11–13^

With changes in chromosomal structure, the biophysical properties of mitotic chromosomes are important for understanding the condensation and decondensation of chromosomes. Particularly, the complex and surprising chromosomal hierarchy of eukaryotes is highly structured and organized in all stages of an organism's life. Therefore, the biophysical properties of each chromosomal component, including DNA, nucleosomes, and chromatin, are essential for the analysis of such hierarchy in chromosomes.^14–16^ Additionally, due to structural complexity and technological limitations,^1,17–20^ the question remains open as to how the human mitotic chromosomes are specifically condensed at each stage, including the interphase, prophase, metaphase, anaphase, and telophase. Although extensive microscopic studies revealed the condensed supramolecular structure of metaphase chromosomes,^1,18,21^ their detailed biophysical characteristics such as surface charge and nanomechanical mapping are still elusive. The stiffness and surface charge of metaphase chromosomes could be related to their condensation and composition. The degree of chromosome condensation changes with the cell cycle.^22^ In this work, as a proof-of-concept study, we focused on metaphase chromosomes, which are the most condensed materials in mitosis.

Chromosome preparation can be classified into several representative methods.^23–25^ The chromosome spread is among the most commonly used methods for chromosomal analysis, for example, karyotyping. To accurately analyse the biophysical characteristics of chromosomes, they must be purified. The removal process of cellular debris on/around chromosomes by treatment with RNase and proteases has been established over several decades, however, many studies have underestimated that procedure for investigating the biophysical properties of metaphase chromosomes.^26,27^ We have demonstrated the removal of cellular debris on the chromosome in a visual and effective manner using atomic force microscopy (AFM).

We extracted metaphase chromosomes from human B lymphocytes to directly measure them using Kelvin probe force microscopy (KPFM) and PeakForce-quantitative nanomechanics (PF-QNM) based on AFM.^28–32^ The results of AFM analysis clearly showed that the extracted chromosomes were covered with extra positively charged surface-covering materials, distinct from the negative charge properties of chromosomes. We investigated the surface charge and nanomechanical properties of cell debris-free chromosomes and probed them in terms of the hierarchical structure of chromosomes and their components such as DNA, nucleosomes, and chromatin.

## Results and discussion

### Chromosome extraction and purification

**Fig. 1:**
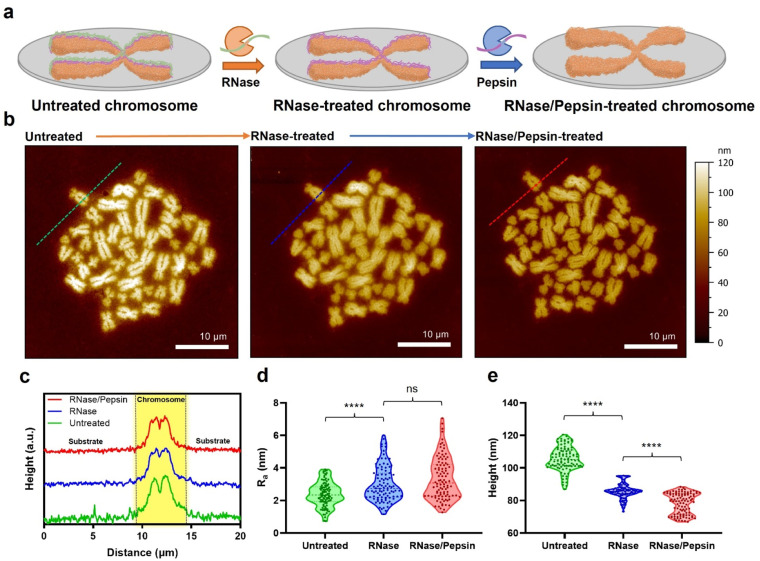
AFM analysis of the hydrolase treatment-based purification process of chromosomes extracted from B lymphocytes. (a) Schematic illustration of the purification of metaphase chromosomes. (b) AFM images of metaphase chromosomes according to enzyme treatment. Data were obtained with the same chromosome set by treating with RNase and pepsin sequentially. (c) Cross-sectional profiles corresponding to the dashed lines in the AFM images in (b). (d and e) Violin plots of average height and surface roughness (*R*_a_) of chromosomes according to enzyme treatment (*n* = 100 per group). The data for the plots were analysed by randomly selecting 100 sites on the chromosomes using MountainsSPIP (version 9) and Gwyddion (version 2.60). Also, the height was measured by randomly selecting the highest 100 points of forty chromosomes, and the roughness was randomly selected by applying the same size of waviness and length. ns: not significant, ****p < 0.0001. A two-tailed unpaired *t*-test was used for statistical analysis.

Metaphase chromosomes extracted from human B lymphocyte cells (RPMI 1788) were immobilized on a freshly cleaved mica substrate (Fig. 1a; see the Materials and methods for details). After obtaining the AFM images of chromosomes, the samples were further treated with hydrolases to remove the extra surface-covering materials on extracted chromosomes. The chromosome sample was incubated with RNase, and subsequently with pepsin. It has been empirically confirmed that if the chromosome is treated with enzymes in reverse order, the removal of surface-covering materials does not work well.^33^ The AFM images of the same sample at each step showed a clear distinction of each chromosome regardless of the enzyme treatment (Fig. 1b). When the samples were examined using AFM phase imaging,^34^ the chromosome samples without hydrolase treatments are indeed covered with blanket-like materials (Fig. S1a†). Although the background cell debris (*e.g.*, RNA and proteins) attached on the flat mica or Si wafer could not be completely hydrolysed by the proteases,^35^ the covering materials on chromosomes seemed to be mostly degraded (Fig. S1b†). All chromosome sampling was performed in the same manner.

AFM data of chromosomes were analysed to confirm the effect of sequential RNase and pepsin treatment. By comparing the cross-section profiles of the same spot (green, blue, and red dashed lines) in each AFM image, it was revealed that as the chromosome was treated with the sequential enzyme treatment, the surface of chromosomes became rougher (Fig. 1c and d). In contrast, interestingly, as the enzymatic treatment progressed, the average roughness of the substrate decreased (Fig. 1d and e and S3†). In a previous work, it was reported that RNA molecules are well adsorbed on the chromosome surface.^35^ As the outermost surface of chromosomes is rough owing to the compact granules, RNA deposition on the chromosome surface conceals the original undulating surface, so making it smoother and flattened.^19^ If the adsorbed RNA could be degraded by RNase, the morphology of the chromosome would return to being rough and undulating. The fact that the surface roughness of chromosomes is only slightly changed after pepsin treatment may be attributed to the small amount of protein on the surface of the chromosomes. Or, by RNase treatment, the adsorbed proteins might be already lifted off together with RNA. As such, both the metaphase chromosome preparation and substrate cleaning were properly done by the sequential enzyme treatment.

### Direct measurement of the surface charge of chromosomes

**Fig. 2:**
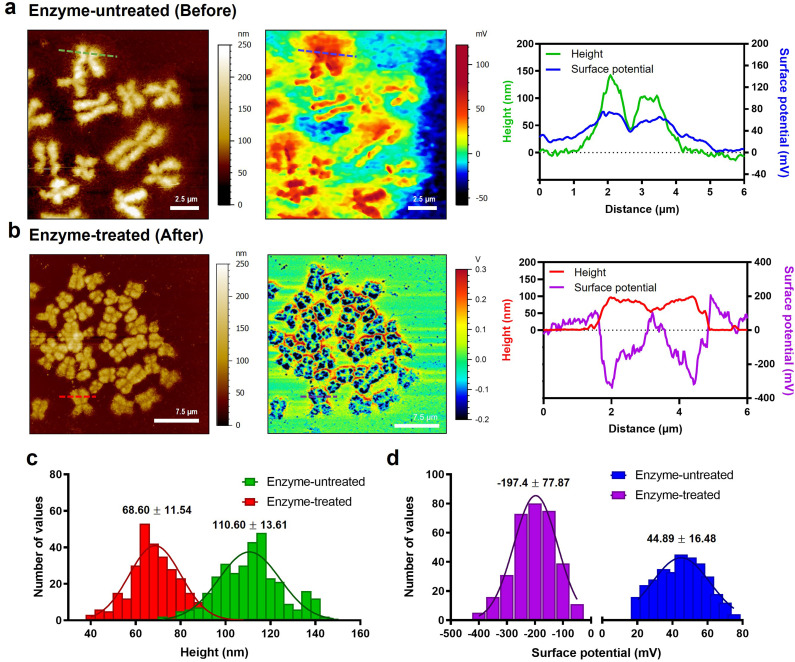
Changes in topography and surface potential of metaphase chromosomes before and after removal of cellular debris by enzyme treatment. (a) AFM topography (left) and KPFM surface potential (middle) images of enzyme-untreated chromosomes. The solid green line in the AFM image and blue line correspond to cross-sectional profiles (right). (b) AFM topography (left) and KPFM surface potential (middle) images of enzyme-treated chromosomes. The solid red line in the AFM image and purple line correspond to cross-sectional profiles (right). (c and d) Histograms of height and surface potential distribution showing the difference between enzyme-untreated and enzyme-treated chromosomes (*n* > 300 per group). The histogram was plotted with height/surface charge values by randomly selecting approximately 200 points from five chromosomes. The data were fitted to a normal Gaussian model, and the mean and standard deviation of best-fit values were calculated.

To check the electrical surface properties of covered and naked chromosomes, we obtained the topography and surface charge maps of the chromosomes using KPFM (Fig. 2a and b; see the Materials and methods for details). KPFM is a nanotechnology that can simultaneously map height and surface potential values for biomolecules under dry conditions.^36^ We confirmed that the metaphase chromosomes used in KPFM analysis were sufficiently dried but not damaged (Fig. S4†).^25^ Remarkably, we observed a more significant difference in the surface potential of enzyme-untreated (covered) (44.9 ± 16.5 mV) and enzyme-treated (naked) chromosomes (−197.4 ± 77.9 mV) (Fig. 2c and d and S5†). Additionally, this is the first visualization result revealing that the net charge of purified metaphase chromosomes, macromolecules highly packed with negatively charged DNA wrapped around a positively charged histone, is strongly negative (Fig. 2b). Our result is consistent with previous literature where chromosomes in an aqueous state move towards the anode on application of an electric field.^37,38^ Although the electrophoretic movement of chromosomes can be used to predict their negative surface charge in the liquid state, it has never been measured directly. The fact that the surface charge of enzyme-treated chromosomes is strongly negative indicates the necessity of the presence of molecules that mediate chromosome condensation.^39^ The analysis of nucleosomes within chromatin fibers^14,18,40^ and metaphase chromosomes using KPFM revealed that they had a strong negative charge (Fig. S6†), indicating that the surface charge of nucleosomes formed by the ionic interaction between the DNA and histone is not neutralized but rather more negative owing to the increased density of DNA. Although condensin is known to provide strong intra-chromosomal condensation in mitotic chromosomes,^41–43^ the exact model for overcoming strong electrostatic repulsion is elusive. It is expected that it will be elucidated using KPFM in future studies.

### Direct measurement of the stiffness of chromosomes

**Fig. 3:**
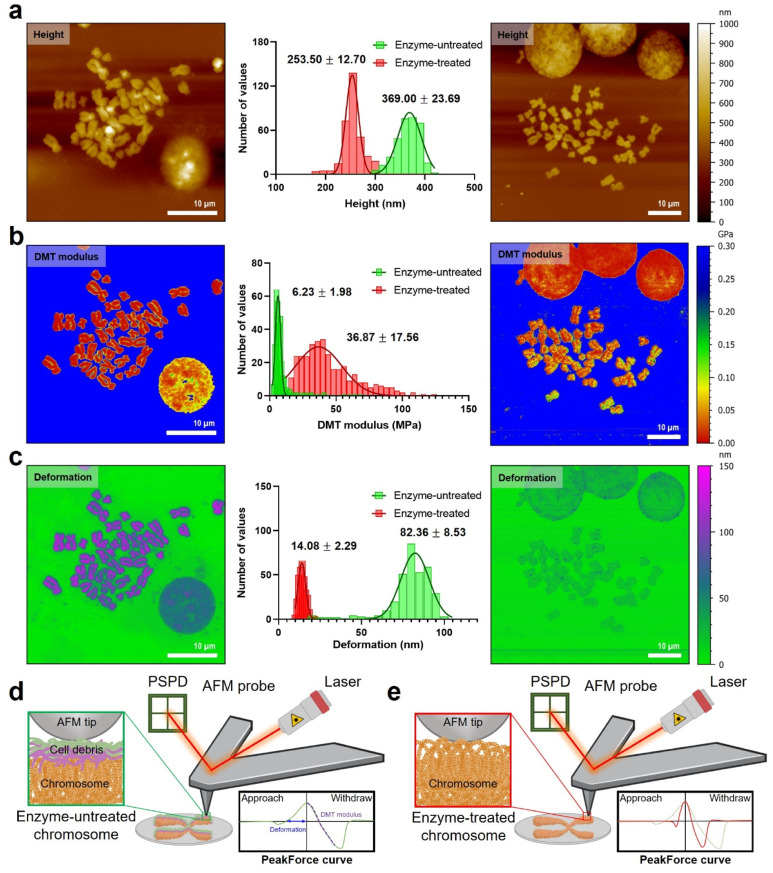
The mapping of nanomechanical properties obtained using PF-QNM in the liquid state for enzyme-untreated (left) and enzyme-treated (right) chromosomes. (a) AFM topography, (b) DMT modulus, and (c) deformation images of enzyme-untreated chromosomes (left) and enzyme-treated chromosomes (right). The distribution histogram (middle) of nanomechanical properties shows the difference according to enzyme treatments (*n* > 300 per group). All histogram distribution data were fitted to a Gaussian model, and the mean and standard deviation of best-fit values were calculated. (d and e) Schematic illustration of the PF-QNM operation for enzyme-untreated (d) and enzyme-treated chromosomes (e). Enzyme-untreated chromosomes have cellular debris whereas enzyme-treated chromosomes do not. The PeakForce tapping cycle reflects the interaction with samples. PSPD: position-sensitive photodiode.

For measurement of the intrinsic stiffness of chromosomes, nanomechanical fingerprints of enzyme-untreated and enzyme-treated chromosomes were investigated using PF-QNM under liquid conditions. Specifically, topography, Derjaguin–Muller–Toporov (DMT) modulus, and deformation were simultaneously mapped under optimal QNM conditions (Fig. 3; see the Materials and methods for details). The mean height of chromosomes in the liquid state was three times higher than that of chromosomes in the dehydrated state, which might be due to water absorption of chromosomes (Fig. S4†). This sponge-like behavior was observed in both enzyme-untreated (from ∼111 nm height for the dehydrated case to ∼368 nm for the hydrated one) and enzyme-treated chromosomes (from ∼69 nm height for the dehydrated case to ∼253 nm for the hydrated one) (Fig. 3a). Interestingly, the height difference between enzyme-untreated and enzyme-treated chromosomes was approximately 1.5-fold in both dehydrated and hydrated cases. This result indicates that the presence or absence of extra materials does not significantly affect the hydration/dehydration characteristics of chromosomes. Additionally, the constant swelling ratio of enzyme-untreated and enzyme-treated chromosomes indicated no damage to chromosomes during enzymatic removal of extra materials, which confirms the reliability of the measured values of nanomechanical properties of chromosomes.

The PF-QNM technique can obtain the DMT modulus of various bio-materials, from soft to stiff ones at high resolution.^44–47^ Note that in the DMT model, the measured stiffness is a value that incorporates adhesion between the AFM tip and samples into Hertzian contact, which is calculated not from the approach force curve but the withdrawal force curve.^48^ Many studies have attempted to investigate the mechanical properties of chromosomes over the past several decades, but most of them relied on indirect measurements.^27,49,50^ Nomura *et al.* tried to directly assess the mechanical stiffness of an enzyme-untreated chromosome using AFM nanoindentation-based force volume mapping (∼160 nm per pixel).^27^ They unveiled the relationship between the chromosome thickness and the elasticity on a single chromosome. What they found was that the elasticity distribution was inhomogeneous over the entire chromosome. In contrast, in our study, we utilized PF-QNM to map the DMT moduli of chromosomes at a high lateral resolution (∼25 nm per pixel).

Our results showed that the difference in the DMT modulus of enzyme-untreated and enzyme-treated chromosomes was significant (Fig. 3b and S7†). Particularly, the DMT modulus map showed a 6-fold increase in the stiffness of enzyme-treated chromosomes (36.9 ± 17.6 MPa) as compared that of enzyme-untreated chromosomes (6.2 ± 1.9 MPa). In the distribution of the DMT modulus histogram and profiles of QNM results, a larger heterogeneity was observed in enzyme-treated chromosomes as compared to that in enzyme-untreated chromosomes (Fig. S7†). Compared to the previous work by Nomura *et al.*,^27^ our chromosome samples are thought to be stiffened owing to either the fixation process with methanol and acetate or an additional dehydration step with ethanol, or both (see the Materials and methods). Our results imply that the chromosomes may be more condensed than intact due to the dehydration process, so the SMC proteins inside chromosomes could be somewhat deformed. However, judging by the fact that the dehydrated chromosomes are rehydrated back under liquid conditions (Fig. S4†), there is probably no severe damage to those proteins in structural maintenance of metaphase chromosomes.

Furthermore, the deformation map demonstrated that enzyme-untreated chromosomes underwent a considerably larger deformation (82.4 ± 8.5 nm) than enzyme-treated chromosomes (14.1 ± 2.3 nm) as shown in Fig. 3c. The result that enzyme-treated chromosomes are harder and less deformed than enzyme-untreated chromosomes was attributable to extra materials consisting of RNA and protein covering the chromosomal surface. In detail, since the thickness of cellular debris (*i.e.*, covering materials) accumulated on the chromosome was measured to be about 100 nm in the liquid state (Fig. 3a and S4†), the deformation around 70 nm in the PF-QNM image is caused by an indentation of cellular debris. In other words, the PF-QNM result implies that the nanomechanical properties of enzyme-untreated chromosomes could be the biophysical properties of cell debris on chromosomes, not the chromosomes themselves. In contrast, the PF-QNM result of the enzyme-treated chromosomes exhibited relatively small deformation values (∼14 nm) for the chromosomes, indicating an indentation of chromosomes in the absence of the cellular debris. Particularly, the nanomechanical maps indicated that the naked chromosomes were structurally more undulating but rigid than chromosomes with debris layers (Fig. 3d and e). All changes in height, surface roughness, surface potential, DMT modulus, and deformation of chromosomes before and after enzyme treatment could be explained by the presence or absence of extra materials consisting of RNA and protein. Taken together, this study suggested that the removal of extra chromosome-covering materials is essential to accurately measure the intrinsic surface charge and nanomechanical properties of naked chromosomes.

### Multiscale stiffness of chromosome organizations

**Fig. 4:**
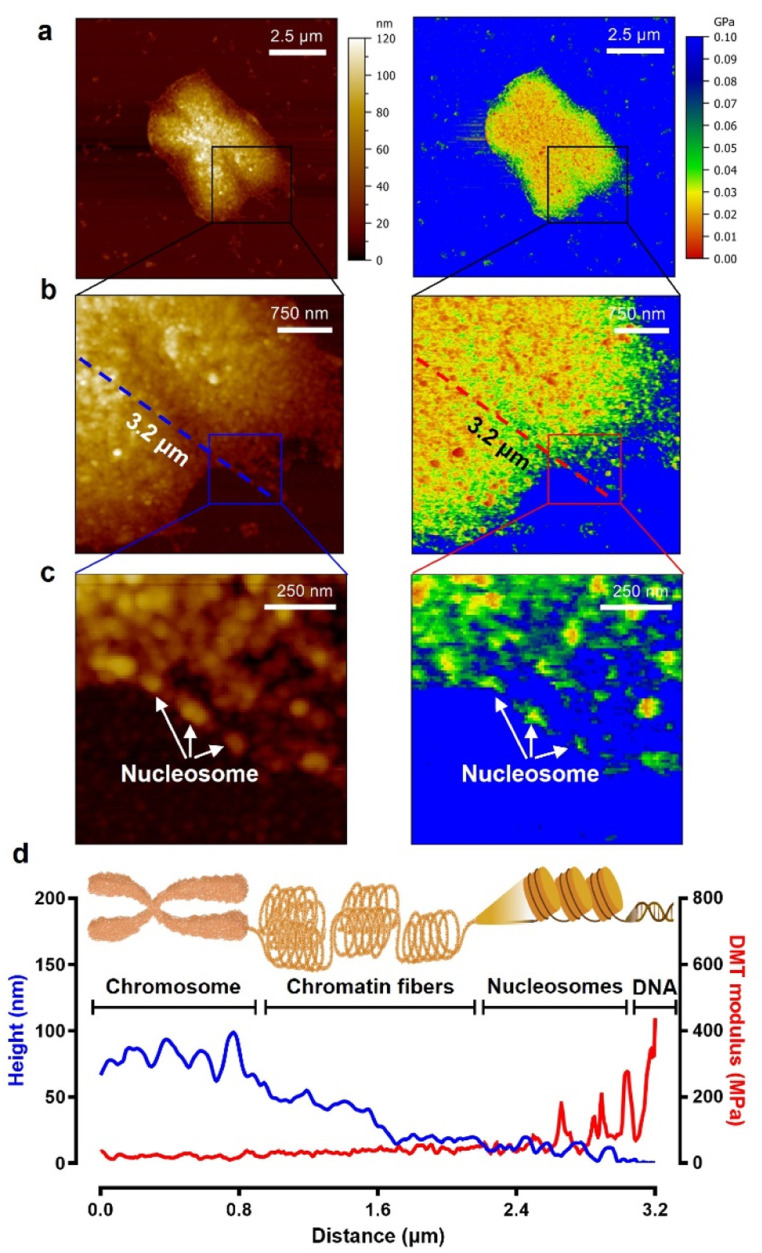
A single chromosome treated with excessive pepsin. (a–c) Topographic (left) and DMT modulus (right) images of a loosened chromosome due to excessive enzyme treatment. (d) The cross-sectional profiles of heights and DMT moduli corresponding to the dashed lines (blue: height, red: DMT modulus) in panel (b). In the hill region of the chromosome edge, chromatin fibers lie between the chromosome body and the nucleosomes.

As seen in the experimental results above, to study the biophysical characteristics of naked chromosomes, the removal of covering materials by enzymatic treatment is very important. However, excessive enzymatic treatment degrades the structural stability of chromosomes.^51,52^ The excessive enzyme-treated chromosomes exhibited significant changes in height and surface roughness (Fig. S8†). Additionally, as shown in the AFM image of Fig. 4, it was observed that the chromosomal structure flowed down from the core to the edge. This phenomenon is problematic for studying metaphase chromosomes, but on the other hand, it is a good opportunity to investigate the hierarchy of chromosome structure. Taking advantage of this, high-resolution PF-QNM was performed focusing on the chromosomal region from the core to the edge in the liquid state (Fig. 4a–c). In the topology and DMT modulus maps of excessive enzyme-treated chromosomes, we identified the hierarchy of chromosomal components such as DNA, nucleosomes, chromatin fibers, and chromosomes (Fig. 4d and S9†). The height of DNA, nucleosomes, chromatin fibers, and chromosomes was 2.10 ± 0.97 nm, 13.46 ± 1.69 nm, 36.33 ± 12.83 nm, and 76.83 ± 4.93 nm, respectively, while their DMT moduli were 236.5 ± 84.1 MPa, 65.2 ± 13.6 MPa, 34.5 ± 5.8 MPa, and 19.1 ± 3.3 MPa, respectively (Table 1). The height and modulus of each part in the chromosome hierarchy were similar to the previous results except for the chromosome.^40,49,50,53^ As quantification in this way could lead to confusion, further work using IR or Raman spectroscopy is required to investigate the structural information of the chromosomal organization more definitively. The chromosomes extracted by us were somewhat stiffer than those reported previously because of the chromosome fixation and dehydration process (Fig. 3; see the Materials and methods). Nevertheless, the measured stiffness values of DNA and nucleosomes making up a chromosome were consistent with those from previous works.^40,53^ In this context, from the PF-QNM measurements, the DMT modulus of chromatin fibers was newly estimated to be approximately 34.5 MPa.

The phenomenon of decreasing stiffness with aggregation of rigid materials to form a complex with hierarchy was due to the internal structural conformation and network. According to previous studies on Young's modulus across single and macromolecules, stiffness tends to decrease as DNA assembles into higher-order complexes from nucleosomes to chromosomes.^54^ For example, the supercoiling structure of chromatin fibers could have a spring-like relaxation effect, and the DMT modulus value gradually decreases as chromosomes are formed. The inverse relationship of DMT modulus according to the hierarchical structure of chromosomes is very similar to that of steel wool.

**Table 1:** Height and DMT modulus of chromosome organizations obtained by PF-QNM (*n* ≥ 30)

Stiffness	DNA	Nucleosomes	Chromatin fibers	Loosened chromosomes^b^	Metaphase chromosomes
Height (mean ± S.D.^a^)	2.10 ± 0.97	13.46 ± 1.69	36.33 ± 12.83	76.83 ± 4.93	253.50 ± 12.70
DMT modulus [MPa] (mean ± S.D.^a^)	236.5 ± 84.1	65.2 ± 13.6	34.5 ± 5.8	19.1 ± 3.3	36.87 ± 17.56

a S.D., standard deviation.

b Excessive enzyme-treated chromosomes (see Fig. 4) were approximately 1.9-fold softer than metaphase chromosomes (36.87 ± 17.56 MPa).

Note that excessive enzyme-treated chromosomes were about 1.9-fold softer than metaphase chromosomes (36.87 ± 17.56 MPa). This means that the ordered structure of chromosomes is somewhat disrupted by excessive pepsin treatment, thereby reducing the condensity. Nevertheless, the stiffness of the loosened chromosomes was more than three times higher than that of the enzyme-untreated chromosomes (6.23 ± 1.98 MPa), suggesting that the internal supercoiling structure was not completely destroyed. Even more surprising is that the stiffness of the chromatin fibers and their supercoiling structure at the edge of loosened chromosomes are quite similar to those of a metaphase chromosome. This result indicates that when chromatin fibers form chromosomes, they are condensed into a fairly compact and ordered structure with the help of the SMC proteins.^55–58^ That is, metaphase chromosomes are not like ordinary steel wool, but like very dense steel wool. Our results indicate that with only the apparent topology of chromosomes, we cannot precisely infer the biophysical properties such as stiffness and/or condensation state of chromosomes, and that the enzyme treatment process to obtain metaphase chromosomes should be delicately considered in the chromosome sampling process.

## Conclusions

In conclusion, we presented the high-resolution mapping of surface charge and stiffness of human metaphase chromosomes using KPFM and PF-QNM. Particularly, the nanoelectrical and nanomechanical properties of chromosomes were altered in the presence or absence of RNA and protein covering materials. Without covering materials, chromosomes exhibited a strong negative charge and stiffness of 36 MPa while they showed a positive charge and stiffness of 6 MPa with covering materials. Details of the material compositions of both covering materials and the positive charge around the chromosomes are still unclear, and could be further analysed using composition mapping technologies, *e.g.*, an IR-AFM combined system.^59^ In this study, the chromosomal components such as DNA, nucleosomes, and chromatin fibers were also analysed with high-resolution PF-QNM, indicating stiffness attenuation of more than 6-fold as DNA condenses into chromosomes. If the composition analysis of the chromosomal components becomes available along with PF-QNM,^59^ it could be possible to further obtain new information throughout the hierarchy of a single metaphase chromosome. Our AFM-based chromosomal analysis revealed that the covering materials distorted the biophysical properties of chromosomes. By utilizing this discovery and technology, we expect to understand the mechanism of chromosomal condensation/decondensation and study the epigenetic instability of chromosomes in cell aging.

## Materials and methods

### Cell culture

RPMI 1788 cell lines were provided by the Korean Cell Line Bank (KCLB, Republic of Korea). RPMI 1788 cells were cultured in RPMI 1640 medium with 2.05 mM l-glutamine (Hyclone, USA) supplemented with 20% FBS (Hyclone, USA) and 1% penicillin–streptomycin (Hyclone, USA).

### Chromosome preparation

Human B lymphocyte cell lines (RPMI1788) were cultured in RPMI 1640 with l-glutamine supplemented with 20% FBS at 37 °C for 72 h under 5% CO_2_ and 95% air. After 78 h, lymphocytes were arrested in metaphase by adding colcemid (Gibco, USA) to the culture medium at a final concentration of 100 ng mL^−1^ for 6–12 h. The cell suspension was then exposed to 75 mM KCl at room temperature for 30 min, and the buffer was then changed to a fixative (methanol : acetic acid = 3 : 1) *via* centrifugation at 300*g* for 10 min. Chromosome spread samples were prepared by dropping the fixed cell suspension solution onto glass, mica, and silicon wafers in a humid atmosphere. Finally, all chromosome samples were dehydrated in a series of ethanol concentrations (Sigma-Aldrich 459836; 70, 80, and 95%) for 2 min before each analysis under AFM. The serial ethanol-drying method is widely used to minimize the deformation artifacts of metaphase chromosomes caused by air-drying.^35^ In our case, ethanol-dried chromosomes are more uniform (*i.e.*, smaller standard deviation in height) in their morphology than air-dried chromosomes (Fig. S2†).

### Enzyme treatment for purification of chromosomes

Chromosome purification was performed *via* sequential enzyme treatment with RNase A and pepsin. Before RNase treatment, the chromosome samples were immersed in 2× SSC (Sigma-Aldrich, S6639) for 3 min. RNase A (Sigma-Aldrich, R6513) stock solution (20 mg mL^−1^) was diluted at 1 : 400 in 2× SSC and applied to the chromosome samples, which were then incubated at 37 °C for 1 h. The slide was then rinsed with 2× SSC at 22 °C for 5 min thrice. Before pepsin treatment, the chromosome samples were rinsed with 10 mM HCl (Sigma-Aldrich, S6639). Pepsin (Sigma-Aldrich, P6887) stock solution (100 mg mL^−1^) was diluted at 1 : 1000 in 10 mM HCl. The chromosome samples were incubated in pepsin solution at 37 °C for 10 min, washed with distilled water (Gibco) for 5 min twice, and dehydrated in a series of ethanol concentrations.

### KPFM

For AFM and KPFM imaging, sampling of enzyme-untreated and enzyme-treated chromosomes was performed on a p-type silicon wafer (ePAK International, USA), which is an electrically conductive substrate. The silicon wafers were immersed in piranha solution (H_2_O_2_ : H_2_SO_4_ = 3 : 1) for 15 min, washed with distilled water, and dried with N_2_ gas (Sejong Industrial Gas Co., Republic of Korea). The chromosomes were examined electrically and topologically using the amplitude-modulated KPFM mode of a MultiMode VIII atomic force microscope (Bruker, USA). KPFM measurements were conducted in the lift scan mode based on the tapping mode at 22 °C under ambient conditions. To measure the nanoelectrical properties of the samples, conductive AFM tips coated with Pt (SCM-PIT-V2; Bruker) were used. In the first scan, a topological AFM image was acquired in the tapping mode with zero-tip bias. In the interleave scan, the AFM tip was lifted 10 nm above the sample surface with an applied sample bias voltage to measure the surface potential. During the interleave scan, the mechanical drive to the cantilever was disabled and an alternating current (AC) bias voltage (*V*_AC_ = 1000 mV) was applied to the probe at mechanical resonance (*ω*) of the cantilever. *V*_AC_ causes the cantilever to oscillate owing to attractive and repulsive electrostatic interactions (*F*_es_) between the probe and sample.
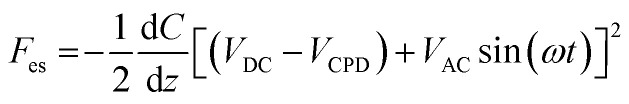
where *V*_DC_ is the direct current (DC) bias voltage and *V*_CPD_ is the contact potential difference between the probe and sample.

A proportional–integral–derivative feedback loop monitors and controls the amplitude of cantilever oscillations by applying compensating *V*_DC_ to the probe to cancel the probe–sample electrostatic forces (*i.e.*, *F*_es_). These depend on the probe–sample capacitance *C* and height *z*. AFM scan conditions were maintained by the amplitude setpoint (6 nm), integral gain (1.0), proportional gain (5.0), and scan rate (0.6 Hz). KPFM images were recorded at 2048 × 2048 pixels. All AFM images were processed line by line, levelled, and analysed using the MountainsSPIP software (version 9; Digital Surf, France).

### PF-QNM in the liquid state

The PF-QNM mode of a MultiMode VIII atomic force microscope (Bruker, USA) was used for mechanical and morphological analyses of chromosomes. PF-QNM measurements were conducted in the peak-force-tapping mode under liquid conditions. For more precise and consistent measurements, the AFM probes (ScanAsyst-Fluid, triangular shape, 0.7 N m^−1^; Bruker) were calibrated on sapphire and polystyrene standards of the calibration kit (Bruker). The chromosomes were incubated in phosphate-buffered saline (pH 7.5) for 30 min and scanned with the PeakForce setpoint (3 nN), feedback gain (10.0), peak force-frequency (2 kHz), amplitude (100 nm), and scan rate (0.8 Hz). The PF-QNM images were recorded at 2048 × 2048 pixels at a frequency of 1 Hz. In the PF-QNM scanning, images were acquired from multiple channels simultaneously, including the height, DMT modulus, adhesion, and deformation.

To obtain Young's modulus, the retract curve was fitted using the DMT model:^60^
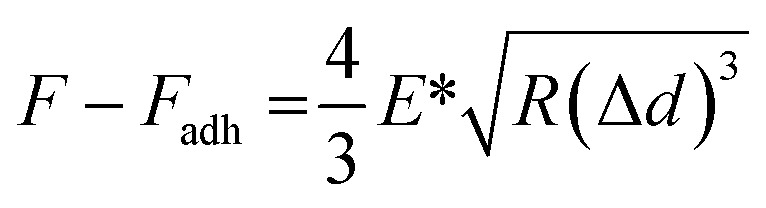
where *F* is the force on the cantilever relative to the adhesion force (*F*_adh_); *R* is the tip end radius; and Δ*d* is the sample deformation. The result of the fit was a reduced modulus. E* was defined as [(1 − *v*_s_^2^)/*E*_s_ − (1 − *v*_tip_^2^)/*E*_tip_]^−1^. If Poisson's ratio (*v*) was known, the software could use this information to calculate the Young's modulus of the sample (*E*_s_).

All parameters were obtained from the cross-section data of chromosomes. To exclude mechanical contributions from nearby hard mica, mechanical properties (DMT moduli, adhesions, and deformations) were extracted from the centre regions of chromosomes. All AFM images were processed line by line, leveled, and analysed using the MountainsSPIP software (version 9; Digital Surf).

### Statistical analysis

Statistical analyses were performed using the GraphPad Prism 8 software (GraphPad Software, USA). Two-tailed unpaired *t*-tests were used to compare the means of the two groups, unless otherwise specified. *P*-values represent the following: ns (not significant), ****p < 0.0001.

## Author contributions

S. R., S. O., and G. L. designed the study; S. R., T. L., and D. Y. C. analysed the data; S. R., T. L., D. Y. C., Y. K., and G. L. collected and curated the data; and S. R., S. O., and G. L. wrote the paper.

## Conflicts of interest

There are no conflicts to declare.

## Supplementary Material

NA-005-D2NA00620K-s001
