# Supplementary material for: Direct observation of surface charge and stiffness of human metaphase chromosomes
Source: Nanoscale Adv. 2022 Dec 20;5(2):368–77. doi: 10.1039/d2na00620k (PMC9846444; doi:10.1039/d2na00620k)
Supplement: NA-005-D2NA00620K-s001 [file NA-005-D2NA00620K-s001.pdf]

Electronic Supplementary Material (ESI) for Nanoscale Advances.
This journal is © The Royal Society of Chemistry 2023

Supplementary Information

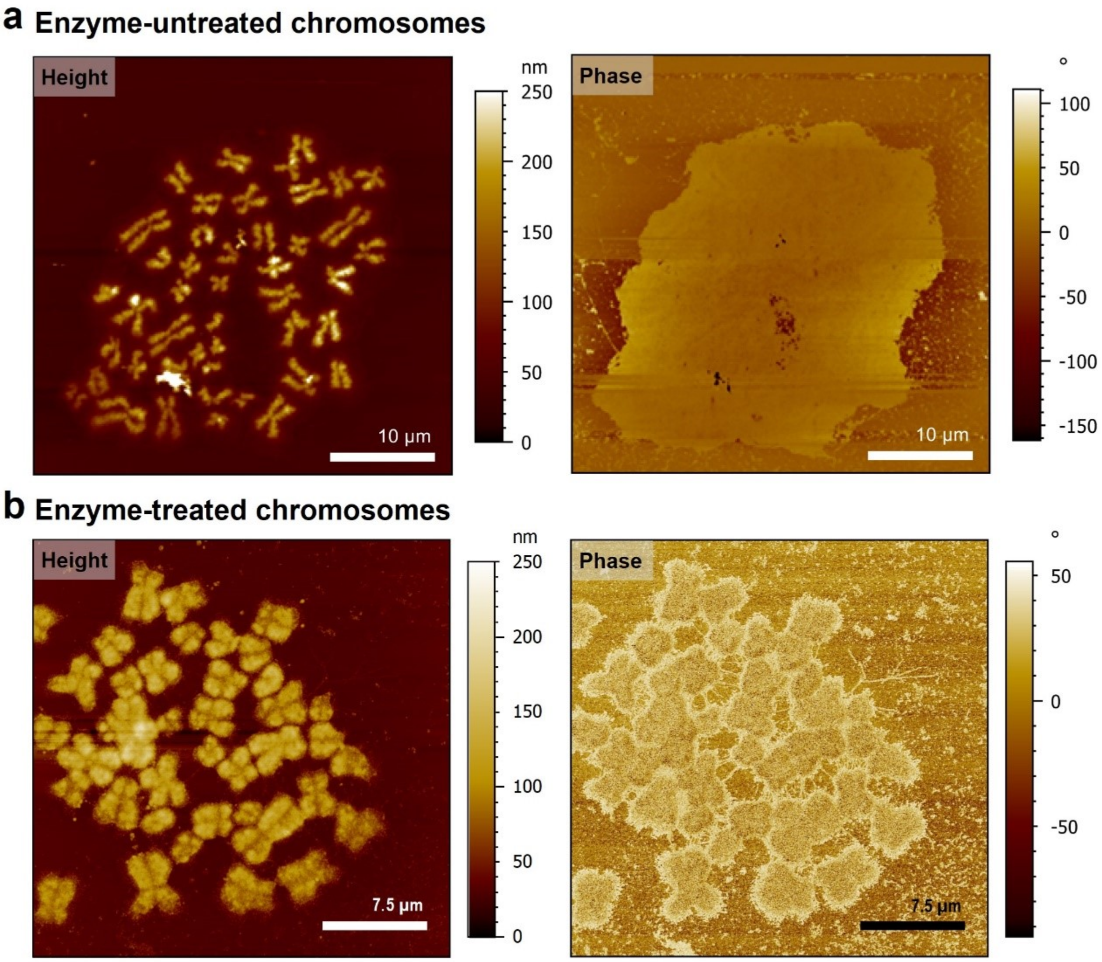

Figure S1. AFM topological and phase images of enzyme-untreated and enzyme-treated chromosomes. a, AFM topological (left) and phase (right) images of enzyme-untreated chromosomes. **b,** AFM topological (left) and phase (right) images of enzyme-treated chromosomes.

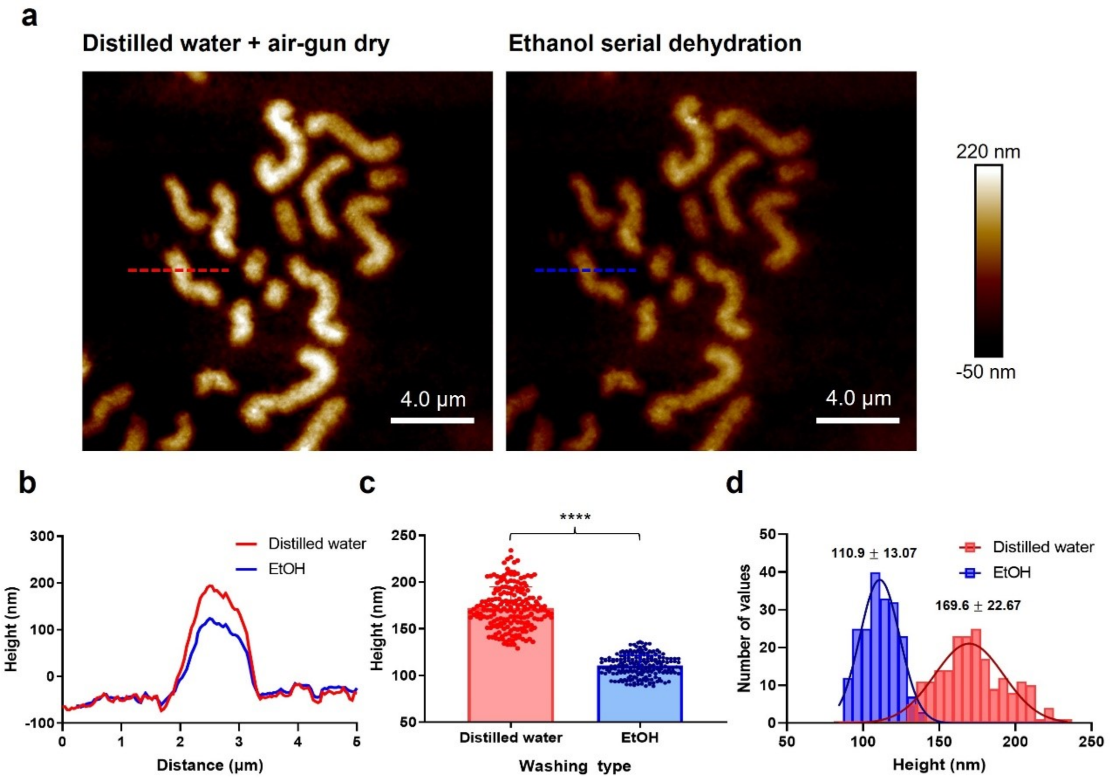

Figure S2. Morphological changes of chromosomes according to EtOH. a, AFM images of chromosome sets fabricated using the two methods. **b,** Cross-sectional profiles corresponding to the dashed lines in AFM images in **a**. **c, d,** Bar graph and distribution of histogram according to drying processing (n = 100 per one group). ****p<0.0001. A two-tailed unpaired t-test was used for statistical analysis. The data were fitted to a Gaussian model, and the mean and standard deviation of best-fit values were calculated.

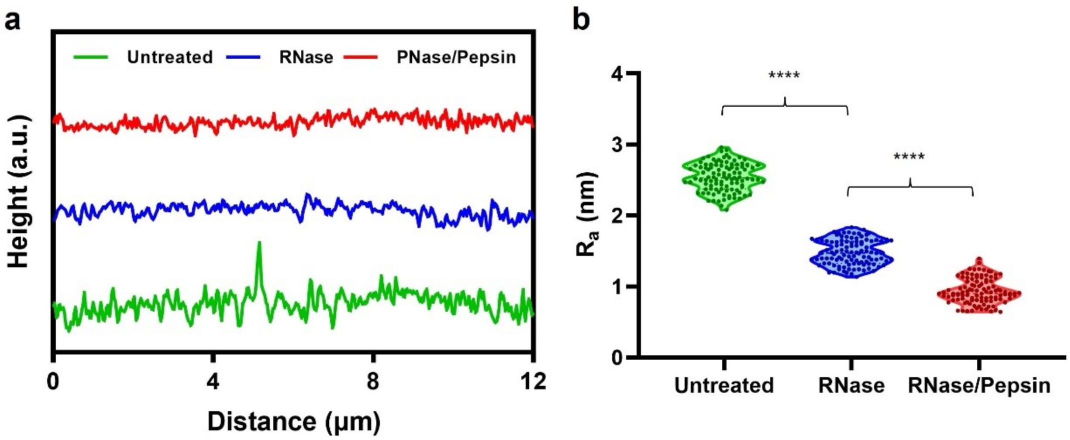

Figure S3. Characterization of substrates near chromosome sample according to enzyme treatment. a, Cross-sectional profiles of substrates according to enzyme treatment **b,** Violin plot of the average roughness of substrates according to enzyme treatment (n = 100 per one group). Roughness average analyses were performed using Gwyddion (version 2.60). ****p<0.0001. A two-tailed unpaired t-test was used for statistical analysis.

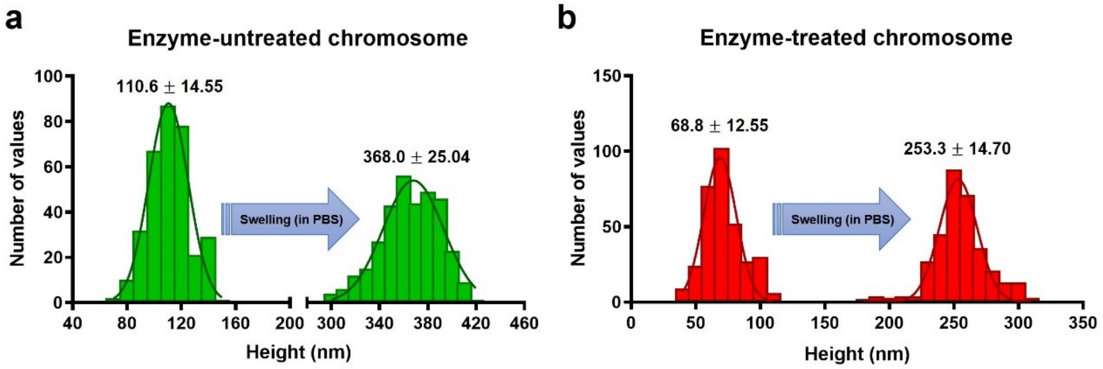

Figure S4. Comparison of the heights between dehydrated and re-hydrated chromosomes. a, Histogram shift of enzyme-untreated chromosomes according to swelling in PBS buffer. The original height was 110.6 ± 14.55 nm, but after immersion in PBS for 30 minutes, it became 368.0 ± 25.04 nm, an increase of approximately 3.3 times. **b,** Histogram shift of enzyme-treated chromosomes according to swelling in PBS buffer. The original height was 68.8 ± 12.55 nm, but after immersion in PBS for 30 minutes, it became 253.3 ± 14.70 nm, an increase of approximately 3.7 times. It implies that the covering effect of extra materials on chromosomal hydration is not marginal.

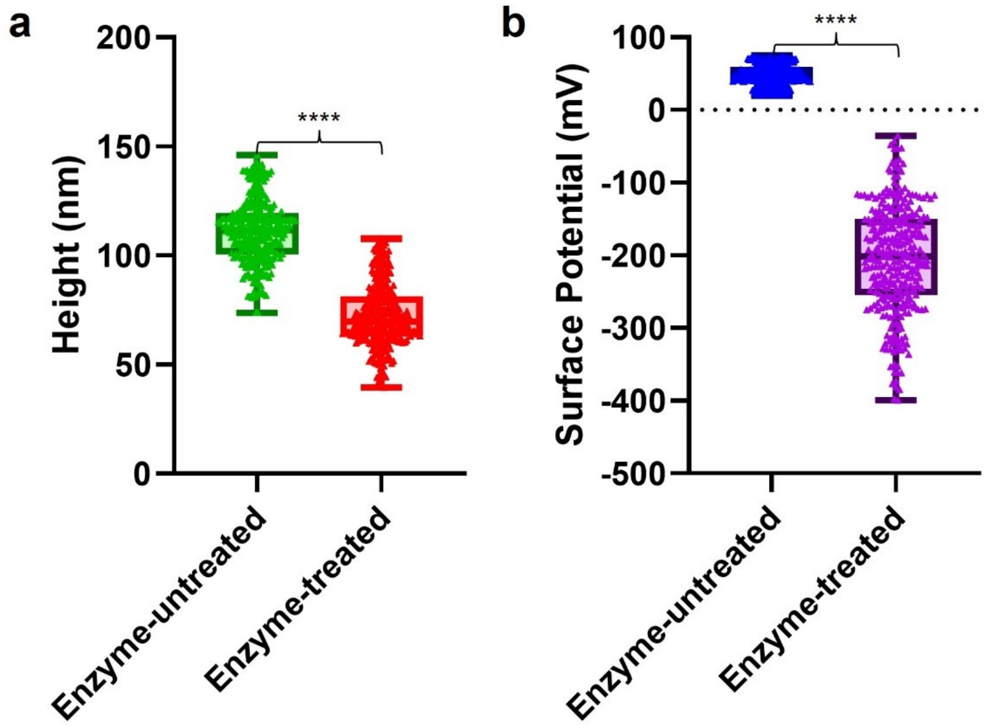

Figure S5. Analysis of electrical properties for enzyme-untreated and enzyme-treated chromosomes. a, Box plots of height and **b,** the surface potential for enzyme-untreated and enzyme-treated chromosomes (n > 300 per one group). ****$p<0.0001$. A two-tailed unpaired t-test was used for statistical analysis.

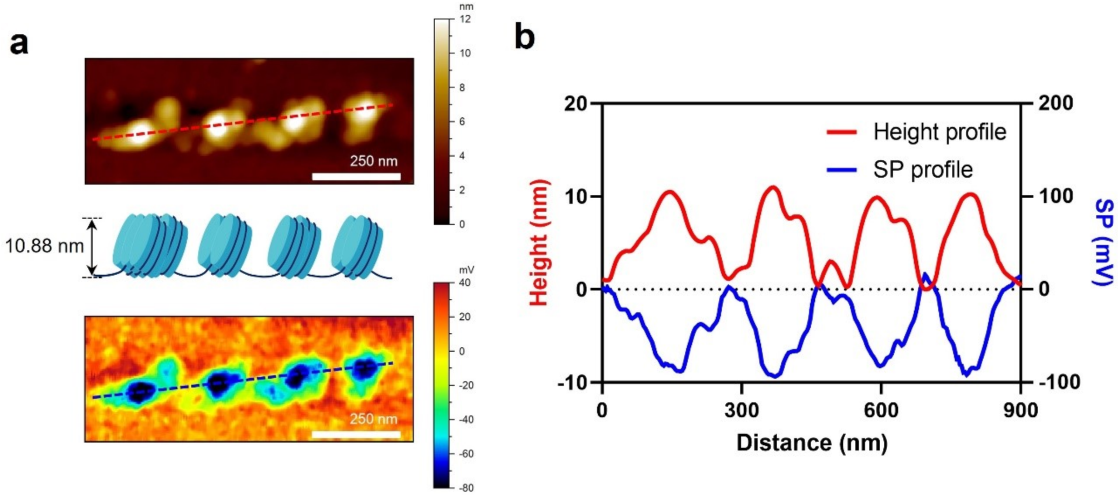

Figure S6. Topography and surface potential characterization of nucleosomes. a, AFM and KPFM map of nucleosomes. **b,** Cross-sectional profiles of height and surface potential corresponding to the dashed lines in AFM and KPFM images in **a**.

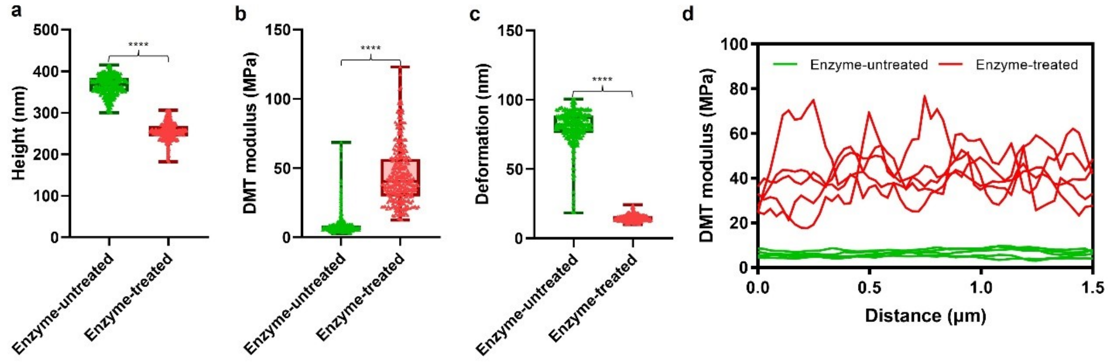

Figure S7. Analysis of nanomechanical properties for enzyme-untreated and enzyme-treated chromosomes. a, b, c, Box plots of height, surface potential, and deformation for enzyme-untreated and enzyme-treated chromosomes (n > 300 per one group). ****p<0.0001. A two-tailed unpaired t-test was used for statistical analysis. These data are taken from Figure 3. **d,** Representative DMT modulus profiles of enzyme-untreated (green curves) and enzyme-treated chromosomes (red curves) from Figure 3b.

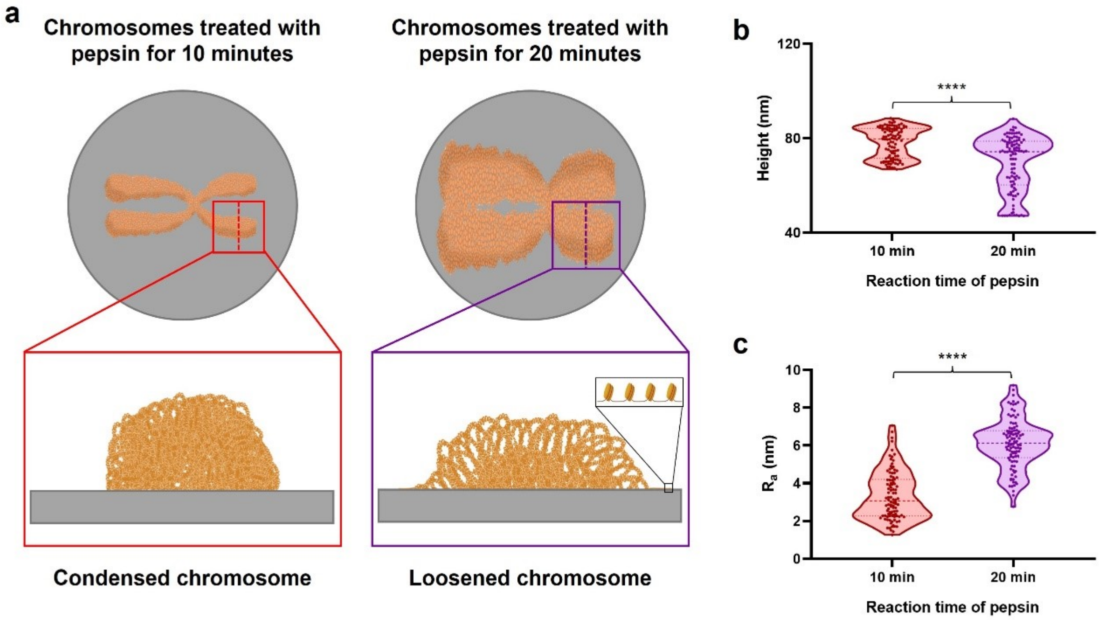

Figure S8. Analysis of differences in height and roughness according to the pepsin exposure time. a, Schematic illustration of properly pepsin-treated chromosomes (10 min) and excessive pepsin-treated chromosomes (20 min). **b, c,** Violin plots of height and average roughness of chromosomes according to pepsin reaction time (n=100 per one group). Average roughness analyses were performed using Gwyddion (version 2.60). ****p<0.0001. A two-tailed unpaired t-test was used for statistical analysis.

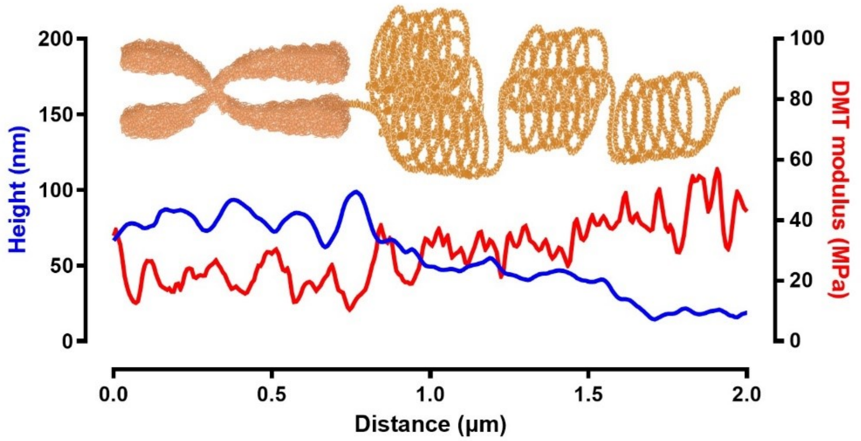

Figure S9. Close-up of chromosome and chromatin fibers in Figure 4d. Corss-sectional profiles of cross-section were obtained in Figure 4b, 4d. Each height and DMT modulus are inversely proportional. Each part of the scheme of chromosomal hierarchy corresponds to cross-sectional profiles according to distance.
